# Cushioned Footwear Effect on Pain and Gait Characteristics of Individuals with Knee Osteoarthritis: A Double-Blinded 3 Month Intervention Study

**DOI:** 10.3390/s23031375

**Published:** 2023-01-26

**Authors:** Isabella Schwartz, Yonah Ofran, Svetlana Bernovsky, Leonid Kandel, Gurion Rivkin, Naama Karniel, Martin Seyres, Sigal Portnoy

**Affiliations:** 1 Faculty of Medicine, Hebrew University of Jerusalem, Jerusalem 91905, Israel; 2 Department of Physical Medicine and Rehabilitation, Hadassah Medical Center, Jerusalem 9765418, Israel; 3 Department of Orthopedics, Hadassah Medical Center, Jerusalem 9765418, Israel; 4 Department of Occupational Therapy, Sackler Faculty of Medicine, Tel Aviv University, Tel Aviv 6997801, Israel

**Keywords:** footwear, osteoarthritis, gait, cushioned shoes, knee pain

## Abstract

One of the recommendations for individuals with knee osteoarthritis (OA) is the use of specific footwear, such as sturdy or cushioned shoes. However, the long-term use effects of using cushioned shoes on the pain and spatiotemporal gait parameters in individuals with knee OA are yet to be reported. We therefore aimed to compare the efficacy of cushioned sport footwear versus sham shoes on motor functions, pain and gait characteristics of individuals with knee OA who used the shoes for 3 months. In a double-blinded study, we provided 26 individuals with knee OA with cushioned sport shoes and 12 individuals with knee OA with similar sport shoes without cushioning for 3 months. The gait analysis, the timed up and go (TUG) test and the Western Ontario and McMaster Universities Arthritis Index (WOMAC) were conducted and the pain levels were measured at the baseline, 1 month, and 3 months after the baseline. We found that the cushioned shoes reduce the amount of pain (based on WOMAC) in the affected knee and increase functionality in the research group, but not in the control group. Gait velocity and cadence were increased in both groups. Gait spatiotemporal parameters and their symmetry were unaffected during the intervention. We conclude that the use of cushioned shoes should be recommended to individuals with knee OA for alleviating pain.

## 1. Introduction

Knee osteoarthritis (OA) is a principal cause of disability in the adult population [1]. Its estimated prevalence in adults above the age of 45 is 27.8%, and in adults above the age of 60, it is 37.4% [2]. The treatment options comprise mainly of weight management [3], exercise [4,5], braces and orthotics [6,7], knee sleeves [8], pharmacologic intervention (e.g., [9]) and surgery [10]. Adjusted footwear is another non-surgical intervention, such as the AposTherapy device [11,12]. The device is a shoe with rails on its plantar area, where two convex shaped elements are attached under the hindfoot and forefoot regions. These elements change the location of the center of pressure during walking, while generating perturbation to challenge neuromuscular control. A clinical trial using the aforementioned device resulted in pain reduction after 8 weeks for the research group, while the control group showed no change in the pain levels. Conversely, there were no significant differences in pain reduction in or improvement to static balance between a group using the Masai Barefoot Technology and the controls following a 12 week period [13]. However, both of the reviewed devices cannot be used during daily activities, but they are provided as an accessory for physiotherapy exercises. Another suggested mean for pain reduction and the prevention of the deterioration of OA is biomechanical footwear, which have been designed for this population.

As reviewed in [14], several studies have incorporated wedged soles and ankle supports. For example, Bennel et al. [15] used shoes with soles of variable stiffness and showed that it can significantly reduce the knee joint load. Rodrigues et [16] showed that a medial insole provides a clinically meaningful decrease in the Western Ontario and McMaster Universities Arthritis Index (WOMAC) scores. Other studies have incorporated comfortable off-the-shelf shoes or various shoes designed specifically for individuals with OA. In a study [17] that provided inexpensive, flexible, non-heeled footwear (Moleca^®^; not designed exclusively for alleviating OA) to older women with knee OA for six months, the research group maintained a natural gait pattern (no increase in hip moment or the ankle inversion angle) compared to the that of the control group. Shakoor et al. [18] used mobility shoes, i.e., flat, flexible, lightweight shoes, designed to imitate barefoot walking. The authors reported gait adaptation resulting in knee load reduction after 24 weeks. However, not all reports favor the special shoes. For example, Hinman et al. [19] provided subjects with OA with shoes with triple-density, variable stiffness midsoles and mild lateral wedge insoles designed to unload the knee. The authors reported no additional benefits for the special shoes to those of the conventional shoes, as both of them provided a similar improvement to the degrees of pain and function.

The literature shows that the efficacy of various soles and shoe designs for alleviating pain and promoting function in individuals with knee OA is controversial and depends on the design of the footwear. However, a survey of 204 individuals with knee OA reported that most of them did not receive professional advice from a podiatrist or physiotherapist regarding their footwear [20]. Furthermore, most of the popular professional recommendations were for sturdy shoes (47%) and sport shoes (40%), and 40% of them advised people to use cushioned shoes [20]. Consequently, these were the three shoe types frequently worn by the subjects. Surprisingly, while cushioned footwear is recommended for this population, publications on the use of a cushioned shoe in individuals with knee OA are scarce. One publication reported different sagittal knee kinematics and kinetics when they were using a cushioned shoe [21]. However, to the best of our knowledge, there are no reports regarding the long-term use effect of using cushioned shoes on the pain and spatio-temporal gait parameters of individuals with knee OA. We therefore aimed to compare the efficacy of cushioned footwear versus sham shoes on the pain and gait characteristics of individuals with knee OA who used the shoes for 3 months.

## 2. Materials and Methods

### 2.1. Population

**Figure 1:**
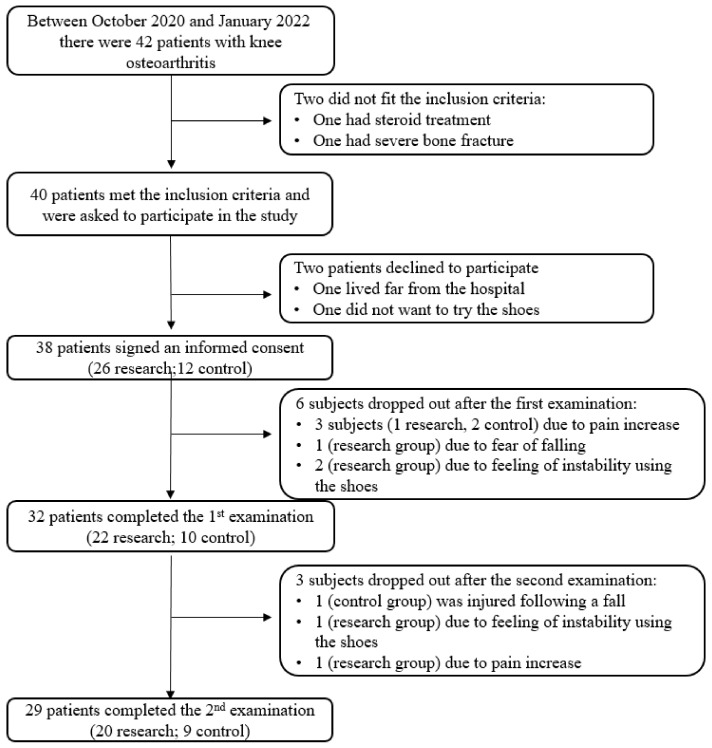
Recruitment process and drop outs during the study.

Thirty-eight individuals fitted the inclusion criteria and signed and informed consent form. The subjects were recruited using a convenient sampling method from a list of patients who were not referred to surgery or declined surgery. The recruitment process and reasons for dropping out are detailed in Figure 1. The inclusion criteria were: men and women aged 45–80, those diagnosed with knee OA (2–4 according to the Kellgren and Lawrence system; [22]), those with a knee pain score of 4–9 according to self-rating on Visual Analogue Scale (VAS; [23]) and those with the ability to walk at least 20 m with or without a walking aid. The exclusion criteria were based on a self-report: those with gait abnormality due to previous orthopedic or neurologic injury, pregnant people and those with open wounds on the plantar area of the foot. The subjects were divided to research and control groups. This was a double-blinded study, as the participants and examiners were not aware of the group allocation. The shoes for the two groups were placed in a random manner in a cupboard. The examiner, who was blinded to the relation of the shoe box to the cushioned or non-cushioned shoes, chose a random shoe box according to the shoe size of the patient and sent the box number to the research coordinator, who had the list that identified if the shoe was a cushioned one or a sham one. The personal details of the participants and various measures at the baseline, which were divided into the research and control groups, are detailed in Table 1. Ten subjects in the research group and three of them in the control group reported ‘0’ pain level in the contra-lateral knee (so that the ipsi-lateral knee in the subjects with bi-lateral OA was the one with higher pain levels, as reported by the subjects). The study was approved by the Hadassah Medical Center Helsinki committee (#0457-18-HMO). All of the subjects read and signed an informed consent form before the trial. The sample size was calculated using G*Power 3.1 (University of Kiel, Kiel, Germany).

### 2.2. Tools

The study tools were as follows: The Kellgren and Lawrence system [24] was used for the classification of osteoarthritis. The rating ranged from ‘0’ (absence of x-ray changes of OA) to ‘4’ (severe OA). The Knee Range of Motion (ROM) was measured using a plastic goniometer. All of the measurements were performed by trained clinicians, who were well practiced in manual goniometry to reduce the bias [25]. Knee pain was rated by the subjects on a VAS, which was 10 cm long, ranging from ‘0’ (no pain) to ‘10’ (maximum imaginable pain). The subjects were asked to rate the pain perceived in each knee in the last 48 h. The minimal clinically significant difference for pain VAS in individuals with knee OA is 1.99 cm [26]. The Western Ontario and McMaster Universities Arthritis Index (WOMAC; [27]) was used to rate the pain (5 questions), stiffness (2 questions) and function (17 questions), which were rated on a VAS. The Minimal Clinically Important Difference (MCID) in the WOMAC for individuals with knee OA were found to be reductions of 8.8 after 2 months of intervention and 6.8 after 6 months of intervention [28]. The timed up and go (TUG) test was utilized for assessing mobility dysfunction, as the subjects were timed while they got up from a chair, walked a 3 m straight line, turned and sat back down on the chair [29].

Spatiotemporal gait parameters were collected using a 10 camera motion capture system (Qualisys, Sweden), which was positioned along a 10 m long path. The path was equipped with a mobile safety harness that can withstand up to 500 kg of a person’s weight and can be fitted to the body characteristics of the subject in order to prevent a fall. Four passive markers were placed bilaterally on the heel and forefoot. The 3D coordinates of the markers and the stance and swing timings which were derived from using the motion capture system were transformed into a code, which was created for this study in LabView (version 2017, National Instruments, Austin, TX, USA). This code also calculates a Symmetry Index (SI) for all of the spatiotemporal parameters according to the following formula [30]:

$$SI=\frac{\left|x_{L}-x_{R}\right|}{\frac{1}{2}\cdot \left(x_{L}+x_{R}\right)}\cdot 100$$

where $x_{L}$ and $x_{R}$ are the values of the spatial or temporal parameters of the left and right legs, respectively. The SI ranges between ‘0’ for perfect symmetry and ‘200’ for complete asymmetry. An increase in the gait velocity of more than 0.048 m/s was considered to be above the minimal clinically significant difference for individuals with knee OA [31]. The minimal clinically important difference is the smallest benefit of value to the subjects, conveying the amount of improvement that is important to the patient and would change their functionality, e.g., an increased number of walking hours per day.

The intervention involved the provision of new sport shoes (LEUK for women and THUN for men; Kybun AG, Roggwil, Switzerland). All of the participants were given the same brand of non-cushioned shoes and the same brand of cushioned shoes. The research group received shoes embedded with a sole that consists of a multi-component polyurethane, in which tiny air bubbles have been sealed. This “air pad” cushioning was designed to promote comfort. The control group was provided with sham shoes, i.e., identical shoes in terms of the exterior, but with regular soles. All of the subjects received bilateral shoes, as provided in most research studies of footwear intervention for individuals with OA [32].

### 2.3. Study Protocol

Before the data collection, each subject was fitted with new shoes according to their group assignment. The subjects were asked to use their assigned shoes as their main footwear for the duration of the trial. Measures from both of the groups were collected at the baseline (recruitment), one month and 3 months after recruitment. All of the measures of knee ROM, pain on the VAS, WOMAC and TUG were collected. In the gait laboratory, after the four markers were placed on the subjects, they were asked to walk at a comfortable speed 3–4 times along the 10 m long paved path. The subjects were asked to rate at the end of the study whether they wore the shoes all of the time, most of the time, a few hours a day or whether they hardly wore the shoes.

### 2.4. Statistical Analyses

Considering an alpha of 0.05, a power of 95% and an allocation ratio of 2:1, we extracted the pain levels reported in [11] for the groups who received the footwear treatment and the controls, and the calculated effect size was 1.577. The calculated samples sizes [33] were 9 and 17 subjects, respectively. For the statistical analysis we used SPSS (Version 28; IBM Corporation). We used the Shapiro–Wilk test to test for the normality of distribution. Most of the data were normally distributed, but we used non-parametric tests since the sample size was small. The between-group differences at recruitment were tested using a Mann–Whitney test. We used the repeated measures analysis of variance (ANOVA) to test for the between-group differences and between the three time points of the examination (when a significance was found, Friedman’s test was used separately for each group using the Wilcoxon post hoc test). The partial η^2^ effect sizes were calculated, and they were interpreted as low, medium or large for values of 0.01, 0.06 and 0.14, respectively [34]. Statistical significance was set at *p* < 0.05.

## 3. Results

**Figure 2:**
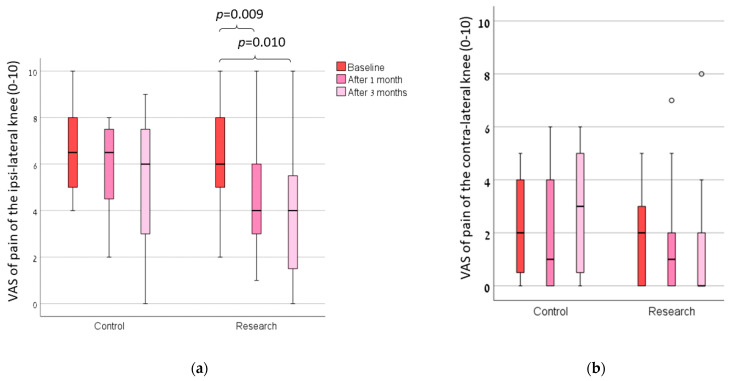
The Visual Analogue Scale of pain in the (**a**) ipsi-lateral and (**b**) contra-lateral knees of both groups at baseline, 1 month and 3 months after the baseline.

**Figure 3:**
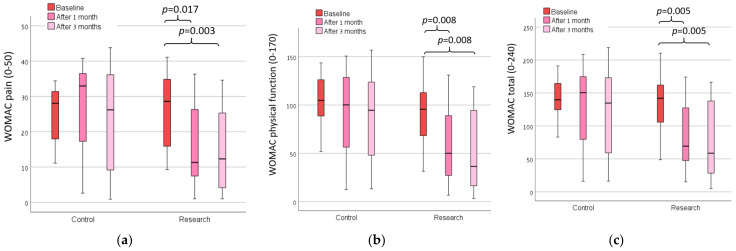
The Western Ontario and McMaster Universities Arthritis Index (WOMAC) for (**a**) pain, (**b**) physical function and (**c**) total score of both groups at baseline, 1 month and 3 months after the baseline.

**Figure 4:**
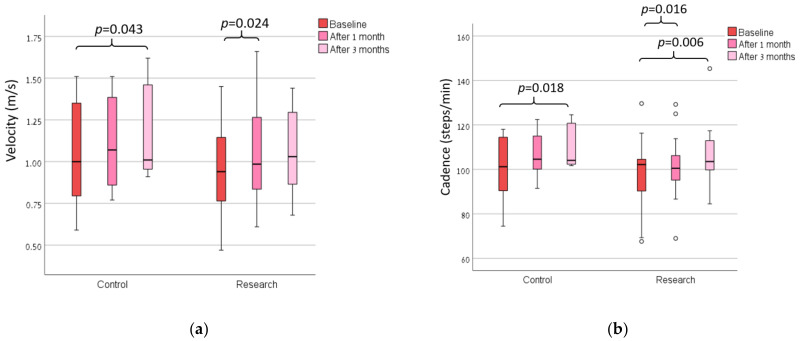
The (**a**) gait velocity and (**b**) cadence of both groups at baseline, 1 month and 3 months after the baseline.

The majority of the participants rated their shoes usage as being between all the time and most of the time. We found no main effect of the group in all of the measures (Table 2; actual numerical data of these measures are presented in a Supplementary Table S1). There was a statistically significant main effect over time for the measures of pain of the ipsi-lateral knee on the VAS (Figure 2), WOMAC (all measures excluding stiffness; Figure 3), gait cadence and velocity (Figure 4). The ANOVA results of these findings are presented in Table 2.

For the VAS of pain score, there were statistical differences in the research group (*p* = 0.005), and there were no statistical differences in the control group (Figure 2). After 3 months, 13 (65%) subjects in the research group and 3 (33.3%) subjects in the control group improved their pain VAS score to that of above the minimal clinically significant difference for individuals with knee OA.

For the WOMAC, there were statistical differences in the research group for the WOMAC pain score (*p* = 0.006; Figure 3a), the WOMAC physical function score (*p* = 0.006; Figure 3b) and the WOMAC total score (*p* = 0.012; Figure 3c).

The within-group differences in gait velocity and cadence are presented in Figure 3. The control group showed a statistically significant improvement in the gait velocity and cadence 3 months after the baseline, whereas there was no improvement in these measures after one month. In the research group, both the gait velocity and cadence improved after one month, and cadence continued to improve two additional months after the previous follow-up. After 1 month, 11 (50%) subjects in the research group and 5 (50%) subjects in the control group increased their gait velocity by more than the minimal clinically significant difference for individuals with knee OA. After 3 months, eight (40%) subjects in the research group and five (55.5%) subjects in the control group increased their gait velocity by more than the minimal clinically significant difference for individuals with knee OA.

In the additional analyses for all of the study population, there was no statistically significant correlation between the ratio of VAS pain score of the ipsi-lateral and contra-lateral knee at the baseline and the WOMAC of physical function score at the baseline (r = −0.233, *p* = 0.285) after 1 month (r = 0.156, *p* = 0.512) and after 3 months (r = 0.066, *p* = 0.787). In the additional analyses on the research group alone, there were no statistically significant differences in the VAS, TUG and WOMAC results between the men (n = 9) and women (n = 17) 1 and 3 months after the baseline examination.

## 4. Discussion

In this double-blinded study, we showed that cushioned sport shoes, which were designed for individuals with knee OA, reduced the amount of pain in the affected knee and increased the functionality, as measured using the WOMAC subcategories. These findings were not significant in the control group. However, velocity and cadence were increased in both the research group, who used the cushioned shoes, and the control group, who used similar un-cushioned shoes. The gait spatiotemporal parameters and their symmetry were unaffected during the long-term (3 months) intervention. While cushioned shoes are sometimes recommended for individuals with knee OA, this is the first piece of evidence of their efficacy in alleviating pain and increasing functionality.

Studies have shown great variability in the progressiveness of knee pain in individuals with OA. The variability is affected by different factors, e.g., knee characteristics, sex (females have worse prognoses) and clinical factors, as well as psychological factors [35]. In our study, 50% of the subjects in the research group experienced a clinically meaningful improvement in the pain levels compared to only 25% of them in the control group, as reported by the pain VAS scores. The WOMAC pain scores show significant reduction of the pain after 1 and 3 months for the research group, but not for the control group. While previous studies using specialized shoes for individuals with OA show pain reduction after 6 months [18] or 3–6 months [19], we show for the first time the knee-pain-alleviating effect of the cushioned shoes in the short term (after only one month). This is an important finding since the persistence knee pain has been found to be predictive of a loss of cartilage volume [36]. Wang et al. [36] suggested that reducing knee pain in individuals with OA in both the early stages of the disease and over time may be important for preserving the knee structure. Following our results, we believe that the cushioned shoes might provide an effective means for this purpose.

The presence of knee pain is a risk factor for reduced physical function. Middle-aged individuals with OA are more likely to develop difficulties in mobility and daily functioning as they become older [37]. A meta-analysis of the literature [35] showed that the presence of bi-lateral knee pain predicts the deterioration of function. Since some of the subjects in our study reported pain in their contralateral limb, we tested for a correlation between the ratio of the VAS pain score of both of the knees (a lower value indicates similar pain levels bilaterally) at the baseline and the WOMAC of physical function score at all three time measurements. Our results do not support the aforementioned prediction. We assume that the reason is that both of our groups were encouraged to walk while wearing their assigned sports shoes (cushioned or not cushioned) so that both of them received the recommended treatment. Possibly another control group that would have continued wearing their own selected shoes would have shown that the deterioration in function can be predicated by the bilateralism of OA.

Although all of the members of the study population increased their gait velocity and cadence, there were no statistically significant differences in the gait pattern or gait symmetry. We expected changes in the gait pattern, in accordance with the previous results exploring the biomechanics of walking with cushioned shoes. In a previous study, the research showed that there are biomechanical differences in older females walking with maximally and minimally cushioned shoes and conventional shoes [21]. Specifically, the authors reported a larger knee adduction moment when the subjects were ambulating while wearing the maximally cushioned shoe. Additionally, the peak knee flexion angle and the loading rates of the vertical ground reaction force were significantly lower when they were using the maximally cushioned shoe [21]. Although the study population did not include individuals with OA, the authors surmised that the reduction of the ground reaction force loading rate when they were using the maximally cushioned shoes might reduce the risk of the deterioration of OA [21]. While the results are encouraging regarding the benefits of using cushioned shoes, we cannot predict that similar results would have been found if an identical study were to be performed in a population with knee OA. This is because it was shown that individuals with knee OA adapt a different walking pattern, both in their gait kinematics [38] and their muscle activation pattern [39], especially when the pain level increases.

The study limitations include a small sample size, although this might not have affected the power of our results, as shown by the large effect sizes presented in Table 2, and also, previous literature concerning footwear efficacy present similar sample sizes [16,18]. Additionally, this sample size exceeds the calculated sample side reported in Section 2.4. Another limitation is that we had no actual record of the number of hours per day that the subjects wore their assigned shoes and the number of hours that the subjects ambulated without wearing the assigned shoes. A third limitation is that we did not measure kinetic parameters that might have provided further insight into the mechanism of gait with the cushioned shoes. Lastly, it should be noted that 3 out of the 26 subjects who received the cushioned shoes felt instability while they were wearing the shoes and dropped out, so this treatment might not fit some individuals with knee OA.

## 5. Conclusions

Our results suggest that using a cushioned sport shoe alleviates pain and improves functionality compared to a regular shoe in individuals with knee OA. Clinicians should therefore recommend using cushioned shoe for this population for the purpose of alleviating pain.

## Figures and Tables

**Table 1:** Personal characteristics of the subjects at baseline. ROM = Range of Motion; VAS = Visual Analogue Scale; TUG = timed up and go; WOMAC = Western Ontario and McMaster Universities Arthritis Index.

	Research Group (N = 26)	Control Group (N = 12)	*p*
Age (years)	67.5 ± 8.8	64.6 ± 8.0	0.171
Sex	9 males, 17 females	8 males, 4 females	0.516
Height (m)	164.4 ± 9.6	167.6 ± 7.6	0.271
Weight (kg)	84.7 ± 15.5	86.5 ± 11.3	0.765
Shoe size (EU)	40.3 ± 2.6	40.8 ± 2.1	0.412
Ipsi-lateral knee	15 right, 11 left	7 right, 5 left	0.330
Kellgren and Lawrence classification (0–4)	2.7 ± 0.7	3.3 ± 0.9	0.078
Flexion ROM of the ipsi-lateral knee (°)	117.1 ± 9.3	118.3 ± 9.4	0.610
VAS ipsi-lateral knee (1–10)	6.5 ±2.1	7.2 ± 2.0	0.381
VAS contra-lateral knee (1–10)	2.4 ± 2.3	2.6 ± 2.5	0.797
TUG (s)	14.1 ± 4.2	13.3 ± 5.2	0.335
WOMAC pain (0–50)	28.4 ± 10.5	23.5 ± 10.4	0.172
WOMAC stiffness (0–20)	12.0 ± 5.7	11.1 ± 5.0	0.649
WOMAC physical function (0–170)	98.8 ± 34.3	95.0 ± 40.3	0.733
WOMAC total (0–240)	141.0 ± 48.2	129.6 ± 52.4	0.451

**Table 2:** Results of the repeated measures analysis of variance (ANOVA). Data are presented as F, p and effect size (partial η^2^). Results of statistical significance are in bold. ROM = Range of Motion; VAS = Visual Analogue Scale; TUG = timed up and go; WOMAC = Western Ontario and McMaster Universities Arthritis Index; SI = Symmetry Index; GC = Gait Cycle.

	Time Effects		Group Effects		Group by Time Interaction	
	*p*	η^2^	*p*	η^2^	*p*	η^2^
Flexion ROM of the ipsi-lateral knee (°)	0.065	0.103	0.659	0.008	0.132	0.078
VAS ipsi-lateral knee (1–10)	0.003	0.206	0.295	0.044	0.586	0.021
VAS contra-lateral knee (1–10)	0.783	0.010	0.324	0.039	0.261	0.052
TUG (s)	0.238	0.058	0.493	0.020	0.698	0.015
WOMAC pain (0–50)	0.044	0.117	0.165	0.076	0.040	0.121
WOMAC stiffness (0–20)	0.080	0.096	0.124	0.092	0.795	0.009
WOMAC physical function (0–170)	0.003	0.213	0.070	0.131	0.379	0.040
WOMAC total (0–240)	0.003	0.203	0.107	0.101	0.234	0.056
Velocity (m/s)	0.005	0.223	0.386	0.036	0.750	0.014
Cadence (steps/min)	<0.001	0.302	0.504	0.022	0.704	0.017
Stance duration of the ipsi-lateral limb (%GC)	0.067	0.121	0.532	0.019	0.298	0.056
Step length of the ipsi-lateral limb (cm)	0.036	0.153	0.291	0.056	0.088	0.114
Base width of the ipsi-lateral limb (cm)	0.901	0.005	0.964	<0.001	0.529	0.030
Stance duration SI (0–200)	0.442	0.038	0.192	0.080	0.927	0.004
Double support duration SI (0–200)	0.193	0.075	0.972	<0.001	0.899	0.005

## Data Availability

Not applicable.

## Supplementary Materials

The following supporting information can be downloaded at: https://www.mdpi.com/article/10.3390/s23031375/s1. Table S1: Median and interquartile range of the outcome measures for each group in each measurement time point.
