# Supplementary material for: Cushioned Footwear Effect on Pain and Gait Characteristics of Individuals with Knee Osteoarthritis: A Double-Blinded 3 Month Intervention Study
Source: Sensors (Basel). 2023 Jan 26;23(3):1375. doi: 10.3390/s23031375 (PMC9920540; doi:10.3390/s23031375)
Supplement: Supplementary file 1 [file sensors-23-01375-s001.zip › sensors-2110352-supplementary.pdf]

Supplementary Table S1: Median and interquartile range of the outcome measures for each group in each measurement time point.
ROM = Range of Motion; VAS = Visual Analogue Scale; TUG = timed up and go; WOMAC = Western Ontario and McMaster Universities Arthritis Index; SI = Symmetry Index; GC = Gait Cycle.

	Control group (N=12)			Research group (N=26)		
	Baseline	After 1 month	After 3 months	Baseline	After 1 month	After 3 months
Flexion ROM of the ipsi-lateral knee (º)	111.5 (110.0-124.8)	120.0 (115.0-123.8)	120.0 (112.5-128.8)	120.0 (112.0-125.0)	123.0 (112.0-125.0)	123.0 (120.0-125.0)
VAS ipsi-lateral knee (1-10)	6.5 (5-8)	6.5 (3.75-7.75)	6 (2.5-7.75)	6 (5-8)	4 (3-6)	4 (1-6)
VAS contra-lateral knee (1-10)	2 (0.25-4.5)	1 (0-4.5)	3 (0.25-5)	2 (0-3)	1 (0-2)	0 (0-2)
TUG (s)	11.8 (9.1-17.1)	11.3 (9.0-14.9)	10.7 (8.9-13.9)	12.5 (10.4-16.1)	11.3 (9.5-15.3)	10.9 (9.4-14.6)
WOMAC pain (0-50)	28.1 (16.4-31.4)	32.9 (16.8-38.2)	26.2 (6.6-36.4)	28.6 (15.1-35.2)	11.3 (7.2-27.5)	12.3 (3.7-26.3)
WOMAC stiffness (0-20)	11.6 (10.2-15.2)	10.7 (5.6-16.5)	10.6 (2.0-16.8)	10.5 (4.1-16)	6.7 (3.2-8.9)	5 (1.6-13.2)
WOMAC physical function (0-170)	104.7 (87.8-132.9)	100.1 (54.2-128.9)	94.5 (35.8-126.0)	95.5 (61.5-113.7)	50.1 (25.8-89.5)	36.5 (16.0-96.6)
WOMAC total (0-240)	139.7 (121.7-172.9)	150.4 (77.4-177.1)	134.7 (44.2-177.1)	142 (102.8-163.3)	69.3 (44.2-134.1)	58.7 (23.3-141.6)
Velocity (m/s)	1 (0.71-1.43)	1.07 (0.81-1.39)	1.01 (0.94-1.56)	0.94 (0.75-1.16)	0.99 (0.83-1.28)	1.03 (0.86-1.31)
Cadence (steps/min)	101.2 (81.8-114.4)	104.6 (96.8-118.8)	104.0 (102.2-121.9)	102.2 (89.2-104.9)	100.5 (94.9-106.6)	103.5 (99.6-113.2)
Stance duration of the ipsi-lateral limb (%GC)	63.03 (61.44-65.74)	63.28 (60.43-64.06)	62.19 (61.13-63.3)	62.28 (61.09-64.1)	61.51 (60.46-63.45)	61.31 (60.39-63.25)
Step length of the ipsi-lateral limb (cm)	58.48 (50.81-71.71)	58.86 (47.49-71.06)	63.95 (55.33-78.38)	54.54 (49.38-68.4)	56.45 (51.89-66.62)	58.01 (51.34-64.18)
Base width of the ipsi-lateral limb (cm)	10 (3.93-13.44)	9.97 (7.59-13.31)	9.34 (6.14-16.77)	8.55 (6.04-11.81)	8.72 (7.76-11.32)	8.27 (6.53-10.73)
Stance duration SI (0-200)	3.7 (0.7-4.7)	2.4 (0.8-3.3)	1.7 (0.9-3.3)	3.15 (2.25-5.13)	3.2 (1.7-4.3)	2.35 (1.5-4.43)
Double support duration SI (0-200)	23.2 (5.4-24.6)	6.5 (3.8-19.6)	8 (4.4-18.6)	9.35 (4.1-24.8)	9.25 (1.53-20)	6.9 (3.38-19.48)
